# Patient Characteristics, Procedural Details, and Outcomes of Contemporary Percutaneous Coronary Intervention in Real-World Practice: Insights from Nationwide Thai PCI Registry

**DOI:** 10.1155/2022/5839834

**Published:** 2022-07-12

**Authors:** Nakarin Sansanayudh, Mann Chandavimol, Suphot Srimahachota, Thosaphol Limpijankit, Pisit Hutayanon, Songsak Kiatchoosakun, Sarun Kuanprasert, Noppadol Chamnarnphol, Siriporn Athisakul, Wirash Kehasukcharoen, Anek Kanoksilp, Worawut Roongsangmanoon, Poj Jianmongkol, Pornchai Ngamjanyaporn, Anuchit Wongphen, Dilok Piyayotai, Worawut Tassanawiwat, Wiwat Kanjanarutjawiwat, Rungroj Krittayaphong, Rapeephon Kunjara Na Ayudhya, Piyamitr Sritara, Wacin Budhari, Ammarin Thakkinstian, Wasan Udayachalerm

**Affiliations:** ^1^Cardiology Unit, Department of Medicine, Phramongkutklao Hospital, Ratchathewi, Thailand; ^2^Division of Cardiology, Department of Medicine, Faculty of Medicine Ramathibodi Hospital, Mahidol University, Phutthamonthon, Thailand; ^3^Cardiac Center, King Chulalongkorn Memorial Hospital, Pathum Wan, Thailand; ^4^Cardiology Unit, Department of Medicine, Faculty of Medicine, Thammasat University, Phra Nakhon, Thailand; ^5^Cardiology Unit, Department of Medicine, Khonkaen University, Mueang Khon Kaen, Thailand; ^6^Cardiovascular Division, Department of Medicine, Faculty of Medicine, ChiangMai University, Mueang Chiang Mai, Thailand; ^7^Cardiology Unit, Department of Internal Medicine, Faculty of Medicine, Prince of Songkla University, Hat Yai, Thailand; ^8^Cardiovascular and Intervention Department, Central Chest Institute of Thailand, Mueang Nonthaburi, Thailand; ^9^Division of Cardiology, Department of Medicine, Faculty of Medicine, Srinakharinwirot University, Watthana, Thailand; ^10^Cardiology Unit, Department of Medicine, Buddhachinaraj Phitsanulok Hospital, Mueang Phitsanulok, Thailand; ^11^Cardiology Unit, Department of Medicine, Chonburi Hospital, Mueang Chonburi, Thailand; ^12^CardiologyUnit, Department of Medicine, Udonthani Hospital, Mueang Udon Thani, Thailand; ^13^Cardiology Unit, Department of Medicine, Sunpasitthiprasong Hospital, Mueang Ubon Ratchathani, Thailand; ^14^Cardiac Catheterization Laboratory at Cardiac Center of Excellence, Phrapokklao Hospital, Chanthaburi, Thailand; ^15^Division of Cardiology, Department of Medicine, Faculty of Medicine Siriraj Hospital, Mahidol University, Phutthamonthon, Thailand; ^16^Department of Medicine, Vichaiyut Hospital, Phaya Thai, Thailand; ^17^Department of Clinical Epidemiology and Biostatistics, Faculty of Medicine Ramathibodi Hospital, Ratchathewi, Thailand

## Abstract

**Background:**

Percutaneous coronary intervention (PCI) practice and outcomes vary substantially in different parts of the world. The contemporary data of PCI in Asia are limited and only available from developed Asian countries.

**Objectives:**

To explore the pattern of practice and results of PCI procedures in Thailand as well as a temporal change of PCI practice over time compared with the registry from other countries.

**Methods:**

Thai PCI Registry is a prospective nationwide registry that was an initiative of the Cardiac Intervention Association of Thailand (CIAT). All cardiac catheterization laboratories in Thailand were invited to participate during 2018-2019, and consecutive PCI patients were enrolled and followed up for 1 year. Patient baseline characteristics, procedural details, equipment and medication use, outcomes, and complications were recorded.

**Results:**

Among the 39 hospitals participated, there were 22,741 patients included in this registry. Their mean age (standard deviation) was 64.2 (11.7) years and about 70% were males. The most common presentation was acute coronary syndrome (57%) with a high proportion of ST-elevation myocardial infarction (28%). Nearly two-thirds of patients had multivessel disease and significant left main stenosis was reported in 11%. The transradial approach was used in 44.2%. The procedural success rate was very high (95.2%) despite the high complexity of the lesions (56.9% type C lesion). The incidence of procedural complications was 5.3% and in-hospital mortality was 2.8%.

**Conclusion:**

Thai PCI Registry provides further insights into the current practice and outcomes of PCI in Southeast Asia. The success rate was very high, and the complications were very low despite the high complexity of the treated lesions.

## 1. Background

Coronary artery disease (CAD) is one of the leading causes of death worldwide [1], including in Thailand, in which CAD deaths accounted for 12.4% of all causes of death [2]. Percutaneous coronary intervention (PCI) is currently the main revascularization modality for these patients [3].

PCI data from clinical registries have emerged as a powerful tool to assess healthcare effectiveness and safety, thus improving quality of care, as well as to inform on the real-world impact of treatment [4–7]. Unlike Western countries, the real-world data of PCI in developing countries are very limited. Only one PCI registry of 4,156 patients was performed in Thailand 13 years ago [8]. Since then, the numbers of PCI have been rapidly increasing, as the government had granted universal healthcare coverage for all Thai populations as well as the increased accessibility to PCI in all areas across the country. In addition, the health national policy also provided more scholarships for Interventional Cardiology Training, leading to an increase in the number of catheterization laboratories nationwide.

These big changes during a decade should provide opportunities to learn about the quality of PCI treatment. Therefore, this PCI registry of Thailand was conducted, which aimed to estimate the failure, complications, and mortality rate of PCI and to assess the trend and temporal changes relative to the previous registry. The data from this registry would provide the most up-to-date PCI practice benchmarks and would be a valuable resource for both regional and international healthcare policymakers.

## 2. Method

Thai PCI Registry is a prospective, multi-center, nationwide study initiated by the Cardiac Intervention Association of Thailand (CIAT) in May 2018. All catheterization laboratories in Thailand were invited and 39 hospitals from all regions across the country voluntarily participated in the registry. All consecutive adult patients aged 18 years or older who received PCIs at these participating centers were enrolled after giving their informed consent. For those who could not give written consent, their relatives who were legal representatives decided on their behalf.

The registry was conducted according to the Declaration of Helsinki and the Ethical Guidelines for Human study. The protocol was approved by the Central Research Ethics Committee of Thailand (CREC) [COA-CREC 006/2018] as well as the local Ethics Committee (EC) if required. The details of the study protocol have been previously published and available on the CIAT website (https://www.ciat.or.th/statement/thai-pci-registry/).

The study began in May 2018 and had completed enrollment in August 2019. All lines of information regarding patient characteristics, procedural details, equipment and medication use, complications, and in-hospital outcomes of the patients were systematically recorded using a specifically developed case record form (CRF). All data were transferred to electronic CRFs (eCRF) by research nurses at local sites. The definition and explanation for each variable were described in the investigator brochure, which was distributed to all 39 sites and was available for download from the website. The investigators and research nurses from all sites participated in several network meetings including the hands-on training workshop for eCRF data collection and input data in computers. In addition, we also provided them technical support via telephone and Line^®^ Application if they had any questions or difficulty in data collection.

The collected data were stored at a central data management unit (DMU), Department of Clinical Epidemiology and Biostatistics, Faculty of Medicine, Ramathibodi Hospital. The data accuracy, quality assurance, and quality control were continuously monitored by the DMU. Regular meetings among the DMU team and PCI registry consortium were organized at least once a month to solve invalid data (e.g., out of possible range values, inconsistency data, etc.). Enquiries had been made to local sites to correct data until the consortium was satisfied.

The maintenance and monitoring of the data were performed by DMU. A site audit was performed at all 39 participating centers. At least 10% of the number of cases at the monitoring time of each site was randomly selected for each site audit. An additional audit was requested by DMU in patients in whom the accuracy of the data was questionable.

The outcomes of interest included PCI success/failure, complications, death in hospitals (all causes of death, cardiovascular death, and specific cause of death), repeated myocardial infarction (MI), repeated revascularization, stroke, heart failure, and bleeding.

## 3. Statistical Analysis

Data including the characteristics of patients, prior morbidities, procedure, and medication use were described using mean or median for continuous data, frequency, and percentage for categorical data. An incidence along with a 95% confidence interval (95% CI) of clinical outcomes (i.e., PCI, success/failure, complications, and death) were estimated. All analyses were performed by STATA version 16.1.

## 4. Results

Of the 39 hospitals participated in the Thai PCI registry, a total of 22,741 patients were included in analyses. The baseline characteristics of these patients are shown in Table 1. The mean (SD) age was 64.2 (11.7) years, approximately two-thirds were 60 years or older, and the octogenarians were nearly 10%, see Table 1. About 70% were male and more than half of all patients were referred cases from other hospitals (54.3%). For healthcare coverage, most patients used universal coverage (62.8%) followed by government services/state enterprises (26.7%). The mean body mass index (BMI) was 24.3 (4.2) with about 60% classified as overweight or obese. About 55% of the patients were either current smokers or ex-smokers. Hypertension and dyslipidemia were reported in approximately two-thirds of the study population. The mean (SD) systolic blood pressure (SBP) and heart rate (HR) at hospital admission were 137.1 (26.8) mmHg and 76.0 (16.7) bpm, respectively. Diabetes and chronic kidney disease (CKD) were found in 44.2% and 32.5%. About one-third and one-fourth of the population had a history of coronary artery disease (CAD) and prior MI, respectively. Prior coronary bypass graft (CABG) and heart failure (HF) were found in 1.6% and 13.8%. History of cerebrovascular accident (CVA) and peripheral arterial disease (PAD) were reported in 5.7% and 1.7%, respectively.

For clinical presentation, most patients presented with acute coronary syndrome (57%) comprised of ST-elevation myocardial infarction (STEMI) 28.0% and non-ST elevation myocardial infarction (NSTEMI) 29.9%. Angiography revealed that single, double, and triple vessel diseases were found in 26.4%, 28.7%, and 33%, respectively, whereas 11% of patients had significant left main coronary artery stenosis.

PCI procedures are described in Table 2. Initial access sites were mainly the transfemoral arteries (53.7%), whereas the transradial approach was 44.2%. Only 1.9% of patients had more than one vascular access approach (e.g., bifemoral access), mainly observed in the complex PCI procedures. Median (range) fluoroscopy time, air kerma, dose area product (DAP) were 12.6 (0.1, 910.0) minutes, 925.0 (80.0, 25810.6) mGy, and 77.4 (20.0, 2939.0) Gy cm^2^, respectively. The median volume of contrast media used was 100.0 (10.0, 600.0) ml. The average number of guiding catheters, guidewires, balloons, and stents required were 1.1, 1.6, 2.0, and 1.5 per procedure, respectively.

Most patients (79.4%) had PCI performed in 1 lesion, whereas the maximum number of treated lesions per patient was 5 lesions (in 9 patients). However, the lesion had very high complexity with most PCI lesions in the registry classified as type C lesion (56.9%) in which intravascular ultrasound (IVUS), optical coherence tomography (OCT), and fractional flow reserve (FFR) were used in 13.9%, 1.0%, and 2.0%, respectively. In addition, intra-aortic balloon pump (IABP) and Rotablator were used in 3.4% and 2.2%, respectively.

The details of medication use are shown in Table 3. Most patients (91.3%) received unfractionated heparin (UFH) as their periprocedural anticoagulant. Aspirin and clopidogrel were mostly prescribed as antiplatelet drugs (i.e., 99.2% and 92.4%, respectively). Other less frequently used P2Y12 inhibitors included Ticagrelor (9%), Prasugrel (1.7%), and Ticlopidine (0.3%).

The outcomes of PCI procedures are described in Table 4. The procedural success rate (95% CI) was as high as 95.2% (94.9%, 95.5%), whereas the procedural complication and in-hospital mortality rate (95% CI) were 5.3% (4.9%, 5.6%) and 2.8% (2.5%, 3.0%), respectively. The most common complication was bleeding [4.8% (4.6%, 5.1%)], in which 237 patients [1% (0.9%, 1.2%)] required blood transfusion. In addition, heart failure, cardiogenic shock during PCI, and myocardial infarction rates occurred in 12.1% (11.7%, 12.6%), 7.9% (7.5%, 8.2%), and 6.1% (5.7%, 6.4%), respectively. Stroke occurred in only 85 patients [0.4% (0.3%, 0.5%)] and nearly two-thirds of them were ischemic stroke. The incidence (95% CI) of in-hospital CABG was 0.3% (0.2%, 0.4%), and the median length of hospital stay was 2 days.

## 5. Trends and Temporal Changes of PCI Practice and Outcomes

The current PCI practice and clinical outcomes have been changed over time when compared to the previous PCI Registry in 2006. For patient characteristics, the current registry had a few years of older patients (64.2 vs 62.7) and had more patients with healthcare universal coverage (63.1% vs 23.7%), current/ex-smoker (55.0% vs 41.4%), history of PCI (29.6% vs 24.7%), chronic renal failure (32.5% vs 6.6%), and diabetes (44.2% vs 37.5%), but had fewer patients with overweight/obesity (60.5% vs 69.7%), previous MI (23.6% vs 29.1%), CABG (1.6% vs 3.9%), PAD (1.7% vs 3.2%), hypertension (67.4% vs 69.1%), and dyslipidemia (65.4% vs 74.7%) relative to the registry 2006 (see Table 1).

Clinical presentations were also different; the current Thai PCI registry contained a higher incidence of STEMI/NSTEMI (57.9% vs 51.3%), cardiogenic shock (8.0% vs 6.2%), and left main disease (11.9% vs 4.5%) relative to the previous registry (see Table 1). Procedures were also changed over time, that is, the current registry had a lower proportion of elective cases (61.2% vs 79.0%) and IABP used (3.4% vs 5.3%) but more radial access (44.2% vs 9.4%) and more use of vascular closure devise (4.8% vs 3.5%) (see Table 2). The trend in antiplatelet drug use had been changed, that is, lower use of Clopidogrel (92.4% vs 96.4%) and Ticlopidine (0.3% vs 7.6%) but increased use of new generation drugs, which were not available in the previous registry in 2006 including Prasugrel (1.7%) and Ticagrelor (9.0%) (see Table 3). Finally, the clinical outcomes of the PCI procedure were improved compared to the previous practice in 2006, i.e., the current practice achieved a higher success rate (95.2% vs 92.5%) (see Table 4).

## 6. Discussion

This Thai PCI Registry is the latest nationwide registry of PCI, which provides contemporary insights into current PCI practice as well as patient demographics, lesion characteristics, device, medication use, and outcomes of PCI in Thailand.

Comparison of the first Thai PCI Registry in 2006 with the current registry in 2018 also revealed important information regarding changes in health system, clinical practice, resource utilization, and clinical outcomes of PCI during the last decade. This information may contribute to a better understanding of the trends in practice, identifying the gaps of knowledge, and leading to improvement in the standard of treatment of the patients.

## 7. Demographic and Patient Characteristics

Compared with other international registries, our patients were as old as PCI patients in Vietnam [9], Korea [10], and Hongkong [11] but much older than those in Malaysia [11] and Singapore [11].

There was a trend for older age of the current Thai PCI patients when compared to the previous Thai PCI registry in 2006. The main increase was observed in the number of patients >80 years old. This reflects the change towards the aging society of Thai population.

More than half of the PCI patients were referred from other hospitals, which reflects the hub and spoke model being used by the Ministry of Public Health of Thailand. It might also reflect the insufficient number of cardiac catheterization laboratories in some areas and the uneven distribution of cardiac catheterization laboratories in the country.

The patients undergoing PCI in Thailand were very high-risk patients. The prevalence of diabetes was 44.2%, which was much higher than the overall prevalence of 9.9% in the general population in Thailand [12]; this number was consistent with the prevalence of diabetic patients from the UK [13], USA [14], Japan [15], and Malaysia [11]. The prevalence of baseline renal insufficiency was also very high (32.5%) and was substantially higher than the prevalence observed in the registry 2006 (6.6%) and other regions from Brazil [16], Australia [17], and USA [18] (23–27%). The other explanation might be the high proportion of STEMI (28%), which was higher than many other registries and the very high rate of cardiogenic shock (8%) in our population.

The proportion of STEMI PCI in Thailand increased from 14% in 2006 to 28%. This could be explained partly because of the successful government policy to establish effective STEMI networks in all areas of the country. The rate of cardiogenic shock before PCI increased from 6.2% in 2006 to 8% in this report. The number was the highest among any national registry of PCI patients. This may be due to the high proportion of STEMI and emergency patients.

Approximately two-thirds of the population had a multivessel disease which was high compared to other registries. Left main disease was reported 11.9%, which was similar to the recent report from Vietnam [19] but was higher than other registries.

## 8. Procedural Details

The initial access site has been changed over time for the use of radial access in Thailand increased from 9.4% in 2006 to 44.2% in 2018-2019. This is in agreement with the trends observed globally. For instance, the ANCALAR registry from Austria reported an increase in radial access from 18.16% in 2011 [20] to 56.57% in 2017 [21]; the CathPCI registry from the United States reported radial access increased from 6.9% in 2010-2011 [18] to 25.2% in 2014 [22] and 44.2% in 2017 [23]. The benefits of radial access over femoral access have been well documented, which is the main reason driving this global trend towards radial first vascular access. Radial access is also endorsed by the national guidelines, but its clinical effectiveness relative to femoral access is still needed to be confirmed using local data from each area around the world. We expect the number of transradial intervention in Thailand to continue to rise in the next decade.

The success rate of initial vascular access was very high (97%). PCI was performed in ACC/AHA classification type C in 56.9%, which is among the highest number reported by other registries. This may be partially explained by the relatively high rate of IVUS used (13.9%) in our population, which was higher than the other international registries with IVUS use <5%, except for the PCI registry from Korea where the IVUS use of 27.5% [10]. One of the main explanations for the high IVUS usage in Thailand is the Public Health policy, which granted full reimbursement of IVUS in all three main healthcare schemes, which covered >97% of PCI patients in this registry. The use of OCT, however, was not as high and was similar to the reports from other PCI registries. This may be due to the limited availability of the device, local expertise, and perhaps the high prevalence of patients with renal insufficiency in this registry.

The use of rotablator in Thailand was quite high (2.15%). Except for Japan (3.7%) [15], other registries reported <2% use of rotablator in PCI patients. The high rate of rotablator use in Thailand was in agreement with the high complexity of treated lesions (more than half of the lesions were type C) as mentioned above.

## 9. Medications

Compared to the registry 2006, we could identify some interesting trends in medication use in PCI patients in Thailand. The periprocedural anticoagulation of choice was UFH in more than 90% of cases with a significant decline in low molecular weighted heparin use. Despite the increase in ACS PCI cases, the use of glycoprotein 2 b/3 a inhibitors substantially decreased, which is in concordance with the current clinical practice guideline recommendations [24].

The CIAT and the Heart Association of Thailand have endorsed the benefit of novel P2Y12 inhibitors over clopidogrel. This is in agreement with all international standard guidelines [24]. However, the use of new P2Y12 inhibitors was only around 10% in Thailand, which due to the price of the new P2Y12 drugs resulted in less accessibility. Ticagrelor was recently listed in the Thailand National List of Essential Medicines in 2018, which enabled it to be reimbursed in all three main healthcare schemes. We expect the use rate of new P2Y12 inhibitors in Thailand to increase in the future. Further, P2Y12 effectiveness based on real-world data should also be assessed.

## 10. PCI Outcomes

Although our PCI patients had high complexity of lesions, the procedural success was as high as 95.2%. The overall complications were 5.3% and bleeding was the most common complication observed (4.8%). The incidence of bleeding from previous registries varied substantially from 0.3% [15] to more than 10% [25] due to the difference in patient characteristics and definition of bleeding. The incidence of blood transfusion was 1% in our registry, which was quite low and comparable with approximately 1-2% observed in other registries [10, 18, 25].

The stroke rate post-PCI remained similar to the incidence in 2006 despite the increase in higher risk patients and more complex lesions. Despite the increase in the number of cardiac surgeons and CABG-available centers across the country, the CABG rate after PCI was 0.3%, which was much lower than 0.8% in 2006. This may reflect the advance in angioplasty technique and more capability of percutaneous intervention as a rescue bailout approach in most PCI centers. This low number may imply that the in-house CABG team should not be mandatory for the set up of new PCI centers as long as the effective CABG referral system to the near center can be established.

The in-hospital mortality rate of PCI in Thailand was 2.8%, which was similar to 2.9% in 2006 despite higher risk patients. The number seems to be higher than many registries which reported an overall in-hospital mortality of approximately 1% [9, 22, 25]. This could be explained by the high number of STEMI and cardiogenic shock patients in our registry. Data from the registry of Melbourne Intervention Group, Australia, with a similar number of STEMI compared to our registry [17] (30.1%), reported in-hospital mortality of 2.3%, comparable with the number observed in our registry.

## 11. Strength and Limitations

This registry was the latest and the largest registry of PCI in Thailand, which is about five times larger than the first registry 2006. In addition, the current registry enrolled more PCI centers of all types (private, government, teaching, and university hospitals) across the country. The results of the registry were better representative of the current practice and outcomes of PCI in Thailand. The completeness and accuracy of the data were outstanding. Unlike some retrospective registries, the data in this registry were prospectively collected using well-constructed CRF and eCRF. The definitions of all variables were standardized and the PI and co-PI from all sites received regular and intensive training. A special web-based data input program was constructed with the focus on detecting errors and inconsistency of data input. A site audit was performed in all 39 participating sites and, quality assurance and quality control were meticulously undertaken. Finally, the project was initiated by CIAT and funded by the National Health System Research Institute, so the registry was independent of any other organization, pharmaceutical, or medical device company.

There were a few limitations in this registry. First, like many PCI registries, the participation was voluntary and not all hospitals in Thailand joined the registry. Fortunately, most of the important PCI centers agreed to participate. According to CIAT survey data, which were the best available data, the total number of PCI in Thailand was approximately 35,000 PCI during the period of the registry. This registry enrolled 22,741 PCI patients, which was estimated to be two-thirds of all PCI in the country. Second, this registry did not collect data of a patient undergoing coronary angiography without PCI; therefore, some lines of information (e.g., percentage of normal coronary angiography, use of FFR in non-PCI patients, and proportion of ad hoc angioplasty) were not available. Third, due to the high burden of the registry on top of the very busy services across the country, the details of some parameters were not collected. Finally, there was no core laboratory for the analysis of Coronary angiogram, and some collected parameters (e.g., lesion length, TIMI flow, and SYNTAX score) were site-reported.

## 12. Conclusion

We have conducted a large-scale nationwide Thai PCI Registry of coronary angioplasty including 22,741 patients, which provides further insights into the current practice and clinical outcomes. The success rate was very high, and the complications were very low despite the high risk of the patient's baseline characteristics and high complexity of treated lesions. The ratios of octogenarian STEMI and cardiogenic shock patients in this registry were higher than other international registries. Furthermore, in accordance with the global trends, there has been a substantial increase in radial access and coronary imaging for PCI in Thailand.

## Figures and Tables

**Table 1:** Demographic and baseline characteristics.

Characteristics	Thai PCI Registry 2018-2019 (*n* = 22,741)	Thai PCI Registry 2006 (*n* = 4,156)
Male gender	15,708 (69.1)	2,877 (69.2)
Mean age (years), mean (SD)	64.2 (11.7)	62.7 (11.3)
<50 years, number (%)	2,630 (12)	
50–59 years, number (%)	5,537 (24)	
60–69 years, number (%)	7,351 (32)	
70–79 years, number (%)	5,124 (23)	
≥80 years, number (%)	2,099 (9)	
Refer case, number (%)	12,350 (54.3)	NA
Payment for PCI, number (%)		
Universal coverage	14,349 (63.1)	983 (23.7)
Civil service	6,106 (26.9)	1,927 (46.4)
Social security service	1,557 (6.8)	173 (4.2)
Self-pay, private insurance, and others	729 (3.2)	1073 (25.8)
BMI (kg/m^2^), mean (SD)	24.3 (4.2)	25.0 (3.9)
Normal (18.5 to 22.99)	7,505 (33.0)	1,115 (26.8)
Underweight (<18.5)	1,474 (6.5)	143 (3.4)
Overweight (23.0 to 24.99)	5,007 (22.0)	940 (22.6)
Obese (≥25)	8,753 (38.5)	1,958 (47.1)
Admission SBP (mmHg), mean (SD)	137.1 (26.8)	NA
Admission HR (bpm), mean (SD)	76.0 (16.7)	NA
Known CAD, number (%)	7,723 (34.0)	NA
Previous MI (>7 days), number (%)	5,366 (23.6)	1,208 (29.1)
Previous PCI, number (%)	6,737 (29.6)	1,028 (24.7)
Previous CABG, number (%)	373 (1.6)	161 (3.9)
Previous CVA/TIA, number (%)	1,296 (5.7)	220 (5.3)
Prior heart failure, number (%)	3,131 (13.8)	NA
Prior valve surgery/procedure, number (%)	101 (0.4)	NA
Chronic renal failure, number (%)	7,398 (32.5)	276 (6.6)
Dialysis, number (%)	814 (3.6)	126 (3.0)
Peripheral arterial disease, number (%)	389 (1.7)	133 (3.2)
Family history of CAD, number (%)	2,058 (9.0)	436 (10.5)
Hypertension, number (%)	15,322 (67.4)	2,870 (69.1)
Dyslipidemia, number (%)	14,862 (65.4)	3,103 (74.7)
Smoking status, number (%)		
Current	5,286 (23.2)	583 (14.0)
Previous	7,239 (31.8)	1,140 (27.4)
Never	10,216 (44.9)	2,423 (58.3)
Diabetes mellitus, number (%)	10,050 (44.2)	1,558 (37.5)

PCI, percutaneous coronary intervention; SD, standard deviation; BMI, body mass index; SBP, systolic blood pressure, HR, heart rate; CAD, coronary artery disease; MI, myocardial infarction; CABG, coronary artery bypass graft; CVA, cerebrovascular accident; TIA, transient ischemic attack; NA, not available.

**Table 2:** Procedural details of Thai PCI Registry.

Variables	Thai PCI Registry 2018-2019 (*n* = 22,741)	Thai PCI Registry 2006 (*n* = 4,156)
Indication for PCI, number (%)		
STEMI	6,373 (28)	581 (14.0)
NSTEMI	6,808 (30)	1,551 (37.3)
Stable CAD	9,562 (42)	2,024 (48.7)
Clinical setting for PCI, number (%)		
Elective	13,926 (61.2)	3,285 (79.0)
Urgent	3,527 (15.5)	409 (9.8)
Emergent	5,288 (23.3)	462 (11.1)
Extent of coronary disease		
1-Vessel	6,011 (26.4)	1,444 (34.7)
2-Vessel	6,529 (28.7)	1,399 (33.7)
3-Vessel	7,495 (33.0)	1,301 (31.3)
Left main stenosis >50%	2,706 (11.9)	187 (4.5)
Access site, number (%)		
Femoral	12,199 (53.6)	3,758 (90.4)
Radial	10,062 (44.2)	392 (9.4)
Brachial	29 (0.1)	4 (0.1)
Combination	433 (1.9)	NA
Other	18 (0.1)	2 (0.0)
More than one attempt for vascular access, number (%)	1,354 (6.0)	NA
Require cross-over of vascular access, number (%)	683 (3.0)	NA
Vascular closure device, number (%)	1,095 (4.8)	144 (3.5)
Number of treated lesions, mean (SD)	1.2 (0.5)	NA
Number of treated lesions, number (%)		
1	18,059 (79.4)	2,692 (64.8)
2	3,925 (17.3)	1,075 (25.9)
3	700 (3.1)	296 (7.1)
4	48 (0.2)	75 (1.8)
5	9 (0.04)	16 (0.4)
6	0 (0.0)	2 (0.05)
Fluoroscopy time, min, median (range)	12.6 (0.1, 910.0)	NA
Air kerma dose, mGy, median (range)	925.0 (80.0, 25810.6)	NA
Dose area product (DAP), Gy.cm^2^, median (range)	77.4 (20.0, 2939.0)	NA
Total volume of contrast, ml, median (range)	100.0 (10.0, 600.0)	NA
Type of contrast used, number (%)		NA
Ultravist	17,069 (75.1)	
Optiray	4,504 (19.8)	
Visipaque	1,241 (5.5)	
Others (Iopamiro, Hexabrix)	24 (0.1)	
Cardiogenic shock before PCI	1,812 (8.0)	257 (6.2)
IABP used, number (%)	772 (3.4)	NA
Other mechanical support, number (%)	14 (0.1)	NA
Number of guiding catheters used, mean (SD)	1.1 (0.4)	NA
Number of guide wire used, mean (SD)	1.6 (1.0)	NA
Number of balloons used, mean (SD)	2.0 (1.4)	NA
Number of stents used, mean (SD)	1.5 (0.9)	NA
Lesion complexity (*N* = 28,246), number (%)		NA
A	1,504 (5.4)	
B1	4,978 (17.8)	
B2	5,617 (20.0)	
C	15,946 (56.9)	
Lesion length, mm, median (range)	24 (1, 100)	NA
IVUS, number (%)	3,161 (13.9)	NA
FFR, number (%)	451 (2.0)	NA
OCT, number (%)	226 (1.0)	NA
Rotablator, number (%)	489 (2.15)	NA

PCI, percutaneous coronary intervention; STEMI, ST-elevation myocardial infarction; NSTEMI, non-ST elevation myocardial infarction; CAD, coronary artery disease; SD, standard deviation; IVUS, intravascular ultrasound; FFR, fractional flow reserve; OCT, optical coherence tomography; NA, not available.

**Table 3:** Medications used Thai PCI Registry.

Cardiac medication	Thai PCI Registry 2018-2019 (*n* = 22,741)	Thai PCI Registry 2006 (*n* = 4,156)
Aspirin	22,362 (99.2)	4,156 (100)
Clopidogrel	20,983 (92.4)	4,007 (96.4)
Ticlopidine	77 (0.3)	314 (7.6)
Prasugrel	390 (1.7)	0^*∗*^
Ticagrelor	2,013 (9.0)	0^*∗*^
Fondaparinux	121 (0.5)	NA
LMWH	2,685 (12.1)	1,384 (33.3)
UFH	20,726 (91.3)	3,475 (83.6)
GP2 b/3 a inhibitors	1,391 (6.3)	615 (14.8)
Home medication^*∗∗*^		NA
ACEI	8,118 (36.7)	
ARB	3,308 (15.0)	
Beta blockers	13,655 (61.7)	
Statin	20,750 (93.8)	
Non-statin lipid drugs	482 (2.2)	
ASA	21,743 (98.3)	
Ticlopidine	100 (0.5)	
Clopidogrel	17,852 (80.7)	
Ticagrelor	3,181 (14.4)	
Prasugrel	636 (2.9)	
Vitamin K antagonist	540 (2.4)	
NOAC	203 (0.9)	

^*∗*^Prasugrel and Ticagrelor were not available in 2006. ^*∗∗*^Percentage of home medication was based on alive at discharge (*N* = 22,115). LWMH, low molecular weight heparin; UFH, unfractionated heparin; Gp2 b/3 a, glycoprotein 2 b/3 a; ACEI, angiotensin-converting enzyme inhibitor; ARB, angiotensin receptor blocker; NOAC, novel oral anticoagulant.

**Table 4:** Outcomes of PCI and in-hospital events.

Clinical outcomes	Thai PCI Registry 2018-2019 (*n* = 22,741)	Thai PCI Registry 2006 (*n* = 4,156)
Procedural success	21,650 (95.2)	3844 (92.5)
Procedural complications	1,199 (5.3)	91 (2.1)
Vascular complications required treatment	69 (0.3)	NA
Bleeding complications	1,102 (4.8)	NA
Bleeding complication requiring transfusion	237 (1.0)	29 (0.7)
Myocardial infarction	1,375 (6.1)	172 (4.1)
Bypass surgery	78 (0.3)	32 (0.8)
Stroke	85 (0.4)	11 (0.3)
Ischemic	52 (61.9)	6 (54.5)
Hemorrhagic	25 (29.8)	5 (45.5)
Tamponade	36 (0.2)	8 (0.2)
Cardiogenic shock	1,785 (7.8)	131 (3.2)
Heart failure	2,758 (12.1)	106 (2.6)
Renal failure	NA	88 (2.1)
New onset of dialysis	119 (0.52)	NA
Death	626 (2.8)	119 (2.9)
Cause of death		
Cardiac death	460 (73.5)	88 (73.9)
Non-cardiac death	166 (26.5)	31 (26.0)

NA, Not available. Values of cells are numbers (%).

## Data Availability

The data used to support the findings of this study were supplied by the Cardiac Intervention Association of Thailand (CIAT) and so cannot be made freely available. Requests for access to these data should be made to Associate Professor Nakarin Sansanayudh, MD, PhD, the Principle Investigator of Thai PCI Registry via e-mail or telephone (+66891130099).

## Abbreviations

ACS: Acute coronary syndrome
BMI: Body mass index
CABG: Coronary artery bypass graft
CAD: Coronary artery disease
CI: Confidence interval
CREC: Central Research Ethics Committee of Thailand
CRF: Case record form
CVA: Cerebrovascular accident
DMU: Data management unit
EC: Ethics committee
eCRF: Electronic case record form
FFR: Fractional flow reserve
HR: Heart rate
IABP: Intra-aortic balloon pump
IVUS: Intravascular ultrasound
LVEF: Left ventricular ejection fraction
MI: Myocardial infarction
NSTEMI: Non-ST elevation myocardial infarction
OCT: Optical coherence tomography
PAD: Peripheral arterial disease
PCI: Percutaneous coronary intervention
SBP: Systolic blood pressure
SD: Standard deviation
STEMI: ST-elevation myocardial infarction.

## Additional Points

*Clinical Perspectives.* Competency in medical knowledge: limited data are available on the practice and outcomes of PCI in developing countries. The authors reported the results of a contemporary prospective PCI registry in Thailand and compared them with results from other national registries. Competency in patient care and procedural skills: the success rate of PCI in this nationwide registry was very high, and the complications were very low despite the high risk of the patient's baseline characteristics and high complexity of treated lesions. The ratios of octogenarian STEMI and cardiogenic shock patients in this registry were higher than other international registries. Furthermore, in accordance with the global trend, there has been a substantial increase in radial access and coronary imaging for PCI in Thailand. Translational outlook: data from the registry provide useful information of real-world clinical practice. Contemporary data regarding the patient characteristics, procedural details, and outcomes of PCI in different areas of the world are encouraged because they are crucial in identifying the gap of knowledge and could lead to improvement in the standard of care of PCI patients.
